# The Heart of the Pituitary

**DOI:** 10.1210/jcemcr/luae036

**Published:** 2024-03-16

**Authors:** Roberto Salvatori, Gary L Gallia

**Affiliations:** Division of Endocrinology and Metabolism, Johns Hopkins University, Baltimore, MD 21287, USA; Pituitary Center Johns Hopkins University, Baltimore, MD 21287, USA; Deparment of Neurosurgery, Johns Hopkins University, Baltimore, MD 21287, USA; Pituitary Center Johns Hopkins University, Baltimore, MD 21287, USA

**Keywords:** pituitary adenoma, cystic degeneration

## Image Legend

**Figure 1.**
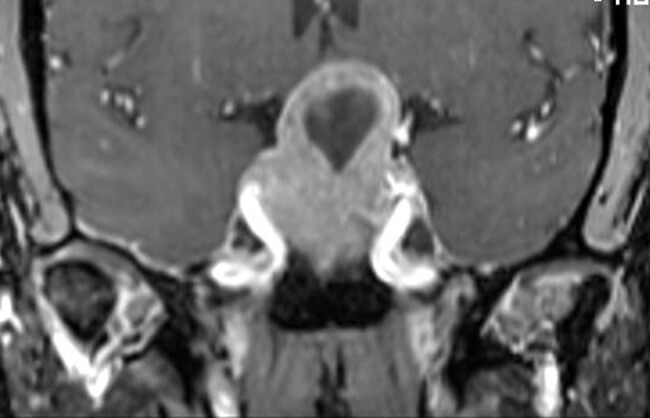
Post gadolinium T1 weighted coronal image of the pituitary mass shows a heart shaped cystic lesion.

A 60-year-old woman presented with bitemporal hemianopsia. A brain magnetic resonance imaging (MRI) scan after gadolinium showed a large sellar/suprasellar mass extending inferiorly along the clivus and compressing the optic chiasm, hypothalamus, and prechiasmatic portions of the optic nerves with a central, heart-shaped, nonenhancing cystic component (Fig. 1). The radiologist thought the findings were most consistent with a craniopharyngioma. Hormonal evaluation showed no evidence of hypersecretory syndromes or hypopituitarism. The patient underwent transsphenoidal surgery, and pathology showed a pituitary adenoma (PA) with immunostains consistent with a gonadotroph adenoma (1). Postoperative MRI showed gross total resection; her anterior and posterior pituitary function remained normal.

Cystic degeneration is seen in nearly half of PAs, thought to be secondary to hemorrhage or infarction that can be silent or manifest as headache of various intensity (2). However, most cystic sellar lesions are Rathke cleft cysts (RCCs) followed by adenomas and craniopharyngiomas. The differential diagnosis of nonfunctioning cystic sellar lesions may be challenging. Some MRI features, such as the presence of intracystic nodules, fluid-fluid level, internal septation, or thick wall enhancement favor the diagnosis of PA, while calcifications (better seen on computed tomography) suggest a craniopharyngioma. The majority of RCCs are round or oval, and off-midline location is uncommon (2).

## Abbreviations

MRI: magnetic resonance imaging
PA: pituitary adenoma
RCC: Rathke cleft cyst
